# 

**Published:** 1908-09

**Authors:** 


### CONTENTS—SEPTEMBER, 1908.—VOL. XXIV, NO. 3.

DEPARTMENT OF PUBLIC HEALTH—

Corpus Christi Meeting of Association of Texas Health Officers .......... 93

ORIGINAL ARTICLES—

The Anatomic Operation for Radical Cure of Inguinal Hernia Under Local Anesthesia. By O. L. Norsworthy, Houston, Texas .......... 100

EDITORIAL DEPARTMENT—

The International Congress on Tuberculosis .......... 111

EDITORIALETS .......... 113

ABSTRACTS AND SELECTIONS .......... 121

BOOKS AND MAGAZINES .......... 130

PUBLISHER'S DEARTMENT .......... 132

